# Efficient Expression of Maltohexaose-Forming *α*-Amylase from* Bacillus stearothermophilus* in* Brevibacillus choshinensis* SP3 and Its Use in Maltose Production

**DOI:** 10.1155/2017/5479762

**Published:** 2017-11-09

**Authors:** Zhu Li, Lingqia Su, Xuguo Duan, Dan Wu, Jing Wu

**Affiliations:** ^1^State Key Laboratory of Food Science and Technology, Jiangnan University, 1800 Lihu Avenue, Wuxi 214122, China; ^2^School of Biotechnology and Key Laboratory of Industrial Biotechnology, Ministry of Education, Jiangnan University, 1800 Lihu Avenue, Wuxi 214122, China; ^3^Department of Food Science and Engineering, College of Light Industry Science and Engineering, Nanjing Forestry University, Nanjing 210037, China

## Abstract

The maltohexaose-forming, Ca^2+^-independent *α*-amylase gene from* Bacillus stearothermophilus* (AmyMH) was efficiently expressed in* Brevibacillus choshinensis* SP3. To improve the production of AmyMH in* B. choshinensis* SP3, the temperature and initial pH of culture medium were optimized. In addition, single-factor and response surface methodologies were pursued to optimize culture medium. Addition of proline to the culture medium significantly improved the production of recombinant *α*-amylase in* B. choshinensis* SP3. This improvement may result from improved cellular integrity of recombinant* B. choshinensis* SP3 in existence of proline. Culture medium optimization resulted in an 8-fold improvement in *α*-amylase yield, which reached 1.72 × 10^4^ U·mL^−1^. The recombinant *α*-amylase was applied to the production of maltose on a laboratory scale. A maltose content of 90.72%, which could be classified as an extremely high maltose syrup, could be achieved using 15% (m/v) corn starch as the substrate. This study demonstrated that the* B. choshinensis* SP3 expression system was able to produce substantial quantities of recombinant *α*-amylase that has potential application in the starch industry.

## 1. Introduction

Alpha-amylase (1,4-*α*-glucan glucanohydrolase; EC 3.2.1.1), which catalyzes the conversion of starch and related carbohydrates into sugar syrups of various types, is used extensively in the pharmaceutical, textile, food, and detergent industries [1]. Each industrial application requires *α*-amylase with specific properties, such as thermostability, substrate specificity, pH and temperature optima, and chelator resistance [2]. As we know, Ca^2+^ helps to stabilize the *α*-amylase currently used in the industrial hydrolysis of starch [3]. As removing this Ca^2+^ from the hydrolysate increases the cost, thermostable *α*-amylase lacking the requirement for Ca^2+^ has been sought [4].

The maltohexaose-forming *α*-amylase from* Bacillus stearothermophilus* (AmyMH) is Ca^2+^-independent *α*-amylase with the potential application in the liquefaction process of starch industry. We recently reported cloning the gene encoding AmyMH, expressing it in* Escherichia coli* BL21 (DE3), and improving its thermostability and Ca^2+^ binding properties through structure-based rational design [2]. However, the industrial potential application of this mutant *α*-amylase is limited due to insufficient yield during protein production.

*Brevibacillus choshinensis* SP3 is a gram-positive bacterium, which has been used for production of several heterologous proteins [5]. The high growth rate, slight level of extracellular proteases, and nonpathogenic capacity make* B. choshinensis* SP3 expression system an excellent host for the recombinant expression of AmyMH. To improve the yield of AmyMH production, fermentation conditions and culture medium were optimized, and then the use of recombinant AmyMH during the maltose production process was initiated.

## 2. Materials and Methods

### 2.1. Bacterial Strains, Plasmids, and Materials

Recombinant* E. coli* BL21 (DE3)/pET-20b-*AmyMH*, which harbors the gene encoding a mutant *α*-amylase (GenBank accession number MG002406) from* Bacillus stearothermophilus* CCTCC WSH13-17, was constructed in our laboratory previously [2].* E. coli* JM109, which was used for cloning work, was purchased from TaKaRa (Dalian, China). The strain* Brevibacillus choshinensis* SP3 and the expression vector pNCMO2 were also purchased from TaKaRa. The constitutive P2 promoter, derived from a cell-wall protein of the host bacterium, is used as the expression promoter for pNCMO2. The following three kinds of recombinant* B. choshinensis* SP3 were used as models.* B. choshinensis* SP3 strains harboring the plasmids pNCMO2/*pal* I were used for the expression of* Serratia plymuthica* sucrose isomerase (NCBI accession number YP_004505648.1) and kept in our laboratory.* B. choshinensis* SP3 strains harboring the plasmids pNCMO2/*cgt* were used for the expression of* B. stearothermophilusα*/*β*-CGTase (PDB: 1CYG_A) and kept in our laboratory.* B. choshinensis* SP3 strains harboring the plasmids pNCMO2/*amyM* were used for the expression of* B. stearothermophilus* maltogenic amylase (GenBank accession number KT337661) and kept in our laboratory.

The DNA Ligation Kit, MutanBEST Kit, polymerase chain reaction reagents, restriction endonucleases, PrimeSTAR HS DNA polymerase, and Agarose Gel DNA Extraction Kit were all purchased from TaKaRa (Dalian, China). Isopropyl *β*-D-1-thiogalactopyranoside, ampicillin, and neomycin were purchased from Sangon Biological Engineering Technology & Services Co. Ltd. All chemicals and reagents were of high quality or analytical grade.

### 2.2. Medium and Cultivation Conditions

For routine construction of plasmids,* E. coli* cells, which harbor the gene of AmyMH, were cultured in LB medium [2] at 37°C with shaking at 200 rpm. The medium and cultivation conditions for the expression of *α*-amylase in recombinant* E. coli* were described in a previous report [2].

The seed medium, which contained 15 g·L^−1^ yeast extract powder, 10 g·L^−1^ glucose, 10 mg·L^−1^ FeSO_4_·7H_2_O, 10 mg·L^−1^ MnSO_4_·4H_2_O, and 1.0 mg·L^−1^ ZnSO_4_·7H_2_O, supplemented with 10 *μ*g·L^−1^ neomycin was used for the seed culture of recombinant* B. choshinensis* SP3. TM medium [6], which contained 10 g·L^−1^ glucose, 10 g·L^−1^ polypeptone, 5 g·L^−1^ meat extract, 2 g·L^−1^ yeast extract, 10 mg·L^−1^ FeSO_4_·7H_2_O, 10 mg·L^−1^ MnSO_4_·4H_2_O, and 1.0 mg·L^−1^ ZnSO_4_·7H_2_O, supplemented with 10 *μ*g·mL^−1^ neomycin was used for the expression of recombinant *α*-amylase in* B. choshinensis* SP3. The seed culture was incubated in a rotary shaker (200 rpm) at 37°C for 12 h. Then, a 5% (v/v) concentration of inoculum was added to TM medium. The resulting medium was incubated for 82 h at 37°C and 200 rpm.

### 2.3. Plasmids Construction

The sequences of the primers used in this study are presented in Table 1. The underlined bases represent restriction sites. To prepare a* B. choshinensis* SP3 expression vector, the AmyMH gene fragment was amplified from pET-20b-*AmyMH* [2] using the primers pNCMO2-F and pNCMO2-R. After restriction analysis utilizing* Pst* I and* Hind* III restriction enzymes and sequencing, the amplicon was ligated into pNCMO2 that had been digested with the same restriction enzymes, yielding the recombinant plasmid pNCMO2-*AmyMH*.

### 2.4. Transformation of* B. choshinensis* SP3

To prepare electroporation-competent cells, an overnight culture of* B. choshinensis* SP3 cultivated in TM medium (37°C, 200 rpm) was diluted 40-fold in TM medium and then grown at 37°C for 4 h. Then, the cells were cooled on ice for 5 min and collected by centrifugation at 5000*g* for 5 min at 4°C. The harvested cells were gently washed four times in cooled SHC buffer (10% sucrose, 16 mM 4-(2-hydroxyethyl)-1-piperazineethanesulfonic acid, 1 mM CaCl_2_, 15% glycerol, pH 7.0). The cells were resuspended in 1/20 volume of SHC buffer for electroporation.

For electroporation, 100 *μ*L of the competent cells was mixed with 100 *μ*L of 15% PEG6000 and 10 *μ*L of recombinant plasmid (100 ng/*μ*L) and then transferred to an electroporation cuvette. The electroporation cuvette was incubated on ice for 10 min and then exposed to a single electrical pulse using a field strength of 21 kV/cm. One mL of TM medium was added to the cells immediately after the electrical discharge. These cells were incubated at 37°C for 2 h and then plated on TM plates containing 30 *μ*g/mL neomycin.

### 2.5. Determination of Biomass

Cell growth was detected by measuring the optical density of the culture medium at 600 nm (OD_600_) [7]. Samples were diluted using pure water to maintain the values within the 0.2–0.8 range and then analyzed using a spectrophotometer (BioPhotometer Plus, Eppendorf Co., Hamburg, Germany). The dry cell weight (DCW) determination was initiated with the centrifugation of 1 mL of culture medium at 13,800 ×g at 4°C for 10 min. The precipitate was washed with pure water and then dried to constant weight at 105°C [6].

### 2.6. Enzyme Assays

Alpha-amylase activity was determined using the method described previously [2]. Sucrose isomerase activity was measured using the method described in a previous report [8]. *α*/*β*-CGTase activity was measured as previously reported [9]. Maltogenic amylase was measured as previously reported [10].

### 2.7. Purification of the AmyMH

The method for purification of AmyMH was described previously [2].

### 2.8. SDS-PAGE and Zymogram Analysis

Sodium dodecyl sulfate-polyarylamide gel electrophoresis (SDS-PAGE) analysis was performed using a previously described method [2]. Zymogram analysis was performed with minor modification of method as previously described [11]. Samples containing AmyMH were separated on a 10% SDS-PAGE gel. The gel was washed with 20 mM sodium phosphate buffer (pH 6.0) at 37°C for 1 h to remove SDS. The washed gel was incubated in fresh buffer containing 1% soluble starch at 70°C for 30 min. This gel was dyed with Lugol solution. Protein bands with *α*-amylase activity became visible as white bands.

### 2.9. Optimization of Enzyme Production

#### 2.9.1. Single-Factor Experiments

The culture conditions and major components of the culture medium, such as temperature, pH, carbon sources, nitrogen sources, and amino acids, were investigated individually to determine their optimal status for the production of AmyMH.

#### 2.9.2. Response Surface Methodology

Central composite design (CCD) was used to analyze the interactions of key factors and the optimal levels of these variables during AmyMH production. In this study, a three-factor, five-level CCD with 19 runs was applied. Each of the tested factors (glucose, yeast extract powder, and proline) was initiated with five different levels (Table 2).

### 2.10. Flow Cytometric Analysis

Samples were harvested during cultivation, washed twice with pure water, and then resuspended in 50 mM phosphate buffer (PBS), pH 7.0, to maintain a cell concentration of approximately 10^5^ cells per mL. These cells were stained with propidium iodide at a final concentration of 0.05 mg·mL^−1^. The test samples were mixed and then incubated at 25°C for 10 min. Flow cytometric analysis was performed with a AccuriC6 COE (BD Biosciences, San Jose, CA, USA). Red fluorescence was captured through a 670 nm long-pass liter. Samples were acquired using a flow rate of 10 *μ*L·min^−1^, with 10000 events being acquired per sample.

### 2.11. Maltose Production

To determine the potential suitability of recombinant *α*-amylase for maltose production from corn starch, experimental maltose production was initiated on a laboratory scale. 15% (m/v) corn starch was suspended in citrate buffer (50 mM, pH 5.5) and gelatinized at 95°C with constant stirring. This starch slurry was liquefied with 90 units of recombinant *α*-amylase or thermostable *α*-amylase (Novozymes®, China) per gram of starch for 30 min. The pH of the hydrolysate was then adjusted to below 4.0 to terminate the reaction. Before the saccharification of the hydrolysate, the pH of the hydrolysate needed to adjust to 5.5. The first-step saccharification of the hydrolysate was initiated by using pullulanase (DuPont™ Genencor® Science, Wuxi, China) and *β*-amylase (DuPont Genencor Science, Wuxi, China) for 6 h at 60°C. The reaction was terminated at high temperature (100°C). The maltogenic amylase was used for second-step saccharification at pH 5.5 and 60°C. The concentration of maltose was analyzed using HPLC method described by Sun et al. [10].

## 3. Results

### 3.1. Expression of AmyMH in* Brevibacillus choshinensis* SP3

**Figure 1:**
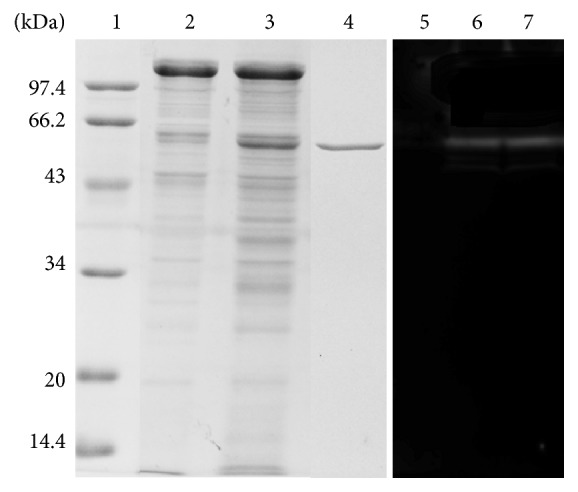
SDS-PAGE with corresponding zymogram of AmyMH expressed in* Brevibacillus choshinensis* SP3.* Lane* 1, molecular mass marker.* Lane* 2, protein from a culture supernatant of* B. choshinensis* SP3/pNCMO2.* Lane* 3, protein from a culture supernatant of* B. choshinensis* SP3/pNCMO2-AmyMH.* Lane* 4, purified samples of AmyMH.* Lane* 5, zymogram of protein from a culture supernatant of* B. choshinensis* SP3/pNCMO2.* Lane* 6, zymogram of the protein from a culture supernatant of* B. choshinensis* SP3/pNCMO2-AmyMH.* Lane* 7, zymogram of the purified AmyMH.

The AmyMH gene from* B. stearothermophilus* was inserted into the expression vector pNCMO2 and then expressed in* B. choshinensis* SP3 in shake-flask cultures. A previously described* E. coli* expression strain [2] in our laboratory was used as a comparator. The *α*-amylase activity produced by recombinant* B. choshinensis* SP3 could reach 2149 U·mL^−1^, with 8.6-fold increase compared to that produced by* E. coli*. The intracellular *α*-amylase activity in* B. choshinensis* SP3 was 143 U·mL^−1^, indicating that the majority of the recombinant AmyMH was secreted into the medium. SDS-PAGE analysis showed that the molecular mass of AmyMH is 56 kDa (Figure 1). A zymogram analysis detected a single band of activity in each lane of the gel (Figure 1). The recombinant AmyMH was purified according to a previously described method [2]. The properties of recombinant AmyMH expressed in* B. choshinensis* SP3 were similar to those of recombinant AmyMH expressed in* E. coli* (Figure S1 and Table S1 in Supplementary Material available online at https://doi.org/10.1155/2017/5479762).

### 3.2. Effect of Temperature and pH on Cell Growth and AmyMH Production

**Figure 2:**
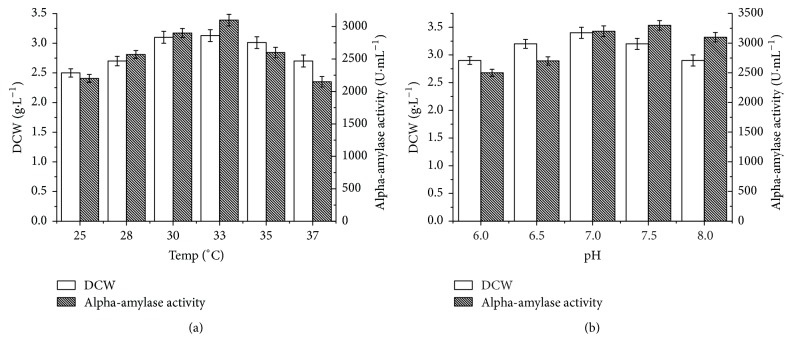
Effects of temperature (a) and pH (b) on dry cell weight (DCW) and *α*-amylase production.

Temperature has obvious influence on microbial fermentation. To optimize the temperature, the recombinant* B. choshinensis* SP3 was cultured at 25, 28, 30, 33, 35, and 37°C. As shown in Figure 2(a), the lowest biomass and *α*-amylase activity were obtained at 25°C, indicating that low temperature was adverse to cell growth and protein expression in* B. choshinensis* SP3. Despite similar biomass at 30, 33, and 35°C, the highest *α*-amylase activity was obtained at 33°C. Thus, the temperature was initiated at 33°C for further study. The influence of different initial pH (6.0, 6.5, 7.0, 7.5, and 8.0) on cell growth and *α*-amylase production was investigated. As shown in Figure 2(b), the initial pH between 6.5 and 7.5 was beneficial for cell growth. The *α*-amylase activity was increased from pH 6.0 to 7.5. When the initial pH raised to 8.0, the *α*-amylase activity was decreased. Therefore, the optimal conditions for expression of *α*-amylase were 33°C and pH 7.5. Under these optimized conditions, the *α*-amylase activity reached 3300 U·mL^−1^, with 1.5-fold increase compared to that obtained under the unoptimized condition.

### 3.3. Single-Factor Optimization of the Culture Medium

**Figure 3:**
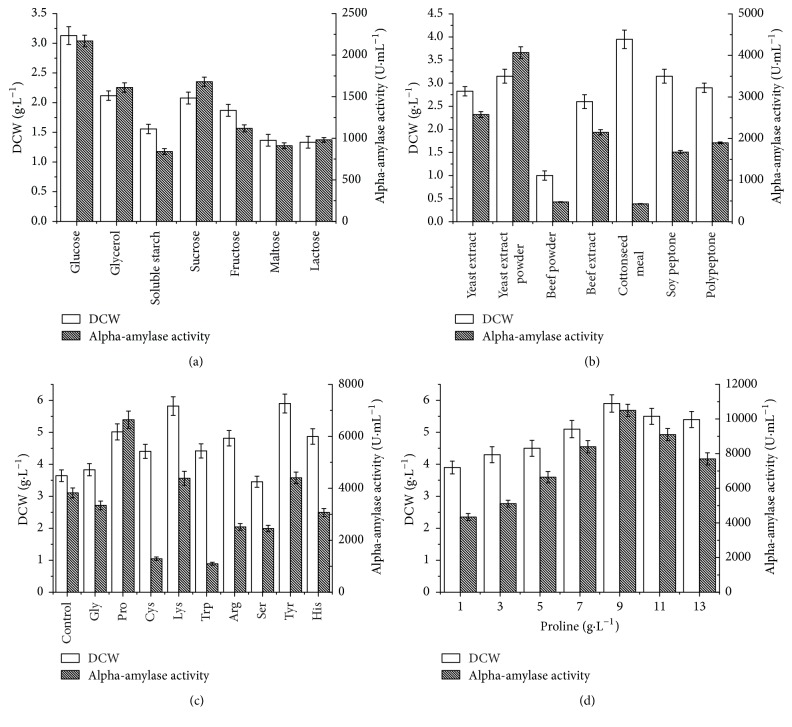
Dry cell weight (DCW) and extracellular *α*-amylase activity obtained from the cultivation of recombinant* B. choshinensis* SP3 using (a) different carbon sources (10 g·L^−1^); (b) different nitrogen sources (10 g·L^−1^); (c) different amino acids (5 g·L^−1^); and (d) different proline concentrations. The DCW and enzyme activity represent the averages of three independent measurements. The error bars represent the standard deviation.

It is well known that microbial amylase production is greatly influenced by the components of their growth medium, especially carbon and nitrogen sources. In order to identify a suitable carbon source, the glucose used in TM medium was replaced with 10 g·L^−1^ glycerol, soluble starch, sucrose, fructose, maltose, and lactose for *α*-amylase production in* B. choshinensis* SP3. The DCW and *α*-amylase production observed using these carbon sources were compared in Figure 3(a). The culture containing glucose as the carbon source exhibited the highest *α*-amylase activity and biomass. In experiments designed to optimize the nitrogen source, the organic nitrogen sources (polypeptone, meat extract, and yeast extract) used in TM medium were replaced with yeast extract, yeast extract powder, beef powder, beef extract, cottonseed meal, soy peptone, and polypeptone, as well as the inorganic nitrogen sources KNO_3_, NH_4_NO_3_, and (NH_4_)_2_SO_4_ at a concentration of 10 g·L^−1^. When inorganic nitrogen sources individually existed in the culture medium, the recombinant* B. choshinensis* SP3 even could not grow in the culture medium (data not shown). Among the organic nitrogen sources, yeast extract powder was the best for *α*-amylase production (Figure 3(b)). Thus, the glucose and yeast extract powder were used in subsequent experiments.

Amino acids can be used as preformed building blocks for protein synthesis or as sole sources of carbon, nitrogen, and energy [12]. In the present study, individual amino acids were added to the medium at a final concentration of 5 g·L^−1^ to investigate their utility in protein production. Addition of most of these amino acids, except for serine and glycine, noticeably enhanced cell growth. However, the addition of glycine, cysteine, tryptophan, arginine, serine, and histidine inhibited *α*-amylase production. Lysine, tryptophan, and proline enhanced *α*-amylase production, with proline having the greatest effect. Since proline was found to significantly enhance AmyMH production, the effects of different proline concentrations on *α*-amylase production were investigated. The results showed that addition of 9 g·L^−1^ proline produces a peak *α*-amylase activity of 1.05 × 10^4^ U·mL^−1^ (Figure 3(d)).

### 3.4. The Effect of Proline on Cell Growth and *α*-Amylase Production

**Figure 4:**
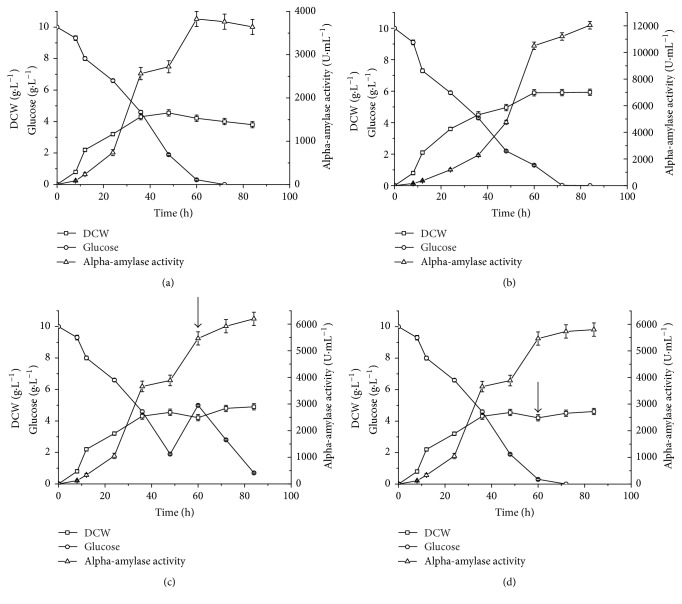
Time profiles of the dry cell weight (DCW), glucose concentration, and extracellular *α*-amylase activity during shake-flask cultivation of recombinant* B. choshinensis* SP3. (a) Without added proline. (b) With 9 g·L^−1^ proline added. (c) Without added proline: arrow indicates the time at which the 5 g·L^−1^ glucose addition was started. (d) With 9 g·L^−1^ proline added when glucose was depleted. The data represent the average of three independent measurements. The error bars represent the standard deviation.

Since the addition of proline significantly increased the production of recombinant *α*-amylase, the probable reasons for this improvement were investigated. As shown in Figure 4(a), when recombinant* B. choshinensis* SP3 was cultured in a medium lacking proline, the DCW reached a peak of 4.5 g·L^−1^ at 48 h and then decreased with time. However, when the culture medium was supplied with 9 g·L^−1^ proline, the DCW could reach 5.9 g·L^−1^ at 60 h (1.3-fold increase compared to that of observed without proline) and remained at 5.9 g·L^−1^ from 60 h to 84 h. The highest yield of *α*-amylase activity without proline addition was 3.82 × 10^3^ U·mL^−1^ at 60 h. When supplied with 9 g·L^−1^ proline, the *α*-amylase activity reached 1.06 × 10^4^ U·mL^−1^ at 60 h (Figure 4(b)) and continued to increase as time went on. The yield of *α*-amylase peaked at 1.20 × 10^4^ U·mL^−1^ at 84 h (Figure 4(b)), with 3.15-fold increase compared to that observed without proline addition. However, the addition of 5 g·L^−1^ glucose or 9 g·L^−1^ proline when glucose was depleted during fermentation produced only a slight improvement in the production of recombinant *α*-amylase (Figures 4(c) and 4(d)).

**Figure 5:**
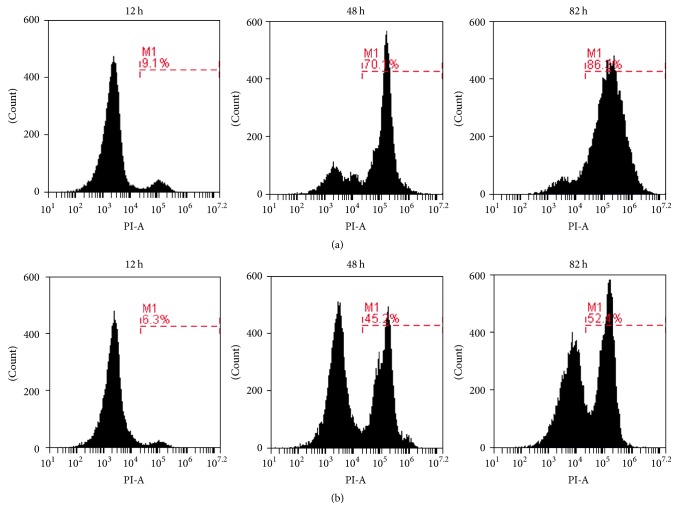
Flow cytometric analysis of recombinant* B. choshinensis* SP3 at different times during the fermentation. (a) Without added proline. (b) With 9 g·L^−1^ proline added.

The integrity and activity of bacterial cells are indispensable for the production of recombinant protein. When the cells are damaged, propidium iodide can bypass the cell membrane and bind to RNA and DNA, causing red fluorescence [13]. Thus, the integrity and activity of the recombinant* B. choshinensis* SP3 cells during fermentation could be evaluated through flow cytometric analysis with propidium iodide staining. The results of a flow cytometric study of* B. choshinensis* SP3 cells cultured with or without added proline are presented in Figure 5. Only a small number of necrotic cells were produced during log phase growth in either culture medium (Figure 5(a), 12 h; Figure 5(b), 12 h). As the incubation time increased to 48 h, the proportion of necrotic cells in the culture medium without added proline increased to 70.1% (Figure 5(a), 48 h). The corresponding value in the culture medium containing 9 g·L^−1^ proline was 45.2% (Figure 5(b), 48 h). Thus, there were far fewer necrotic cells when proline was added to the culture medium. Furthermore, despite the lack of glucose at 82 h during fermentation (Figure 4(b)), the proportion of necrotic cells was only 52.4% when the culture medium was supplemented with 9 g·L^−1^ proline (Figure 5(b), 82 h). In the absence of added proline, 86.3% of cells were damaged and inactive at the same time (Figure 5(a), 82 h). These results suggest that proline helps maintain the integrity and well-being of the recombinant* B. choshinensis* SP3 cells during fermentation. Thus, the enhancement of production of recombinant proteins in* B. choshinensis* SP3 by proline addition might be a universal phenomenon.

To verify that the enhanced production of recombinant proteins caused by proline addition might be a universal phenomenon, another three foreign proteins (maltogenic amylase from* B. stearothermophilus*, sucrose isomerase from* Serratia plymuthica*, and *α*/*β*-CGTase from* B. stearothermophilus*) were expressed by* B. choshinensis* SP3 in medium with added 9 g·L^−1^ proline or a control medium without proline addition. As shown in Table 3, the supplementation of proline slightly stimulated cell growth and improved protein expression in different levels. When culture medium is supplemented with proline, the activities of maltogenic amylase, sucrose isomerase, and *α*/*β*-CGTase increased by 77.1%, 410%, and 53%, respectively. So, the addition of proline might improve the expression of other foreign proteins in* B. choshinensis* SP3 as well.

### 3.5. Optimization of Significant Nutrients Using RSM

To further optimize *α*-amylase production, the three key nutrients (glucose, yeast extract powder, and proline) were simultaneously optimized using response surface methodology. Each of the tested factors was initiated with five different levels. Table 2 shows the actual values, coded factor levels, and corresponding results. The data was analyzed using multiple regression analysis. In addition, the dependence of *α*-amylase production (*Y*) on the levels of glucose (A), yeast extract powder (B), and proline (C) was derived with a quadratic model as follows:

(1) Y=16887.02+622.37A−632.43B+664.36C+8.88AB−545.49AC−1216.13BC−3266.72A2−2874.10B2−2991.58C2.

ANOVA was used to check the adequacy of the model. The *F* value of 42.72 and the *P* value of Prob > *F* < 0.0001 showed the significance of model (Table 4). Linear terms of proline, yeast extract powder, glucose concentrations, and their respective quadratic terms were significant for *α*-amylase production. Among interactive terms, only the interaction of yeast extract powder with proline was significant for *α*-amylase production. The predicated and the experimental values are in good conformity, as the coefficient of determination (*R*^2^) for *α*-amylase production was 0.9771. The signal to noise ratio was 17.284, indicating an adequate signal. The low coefficient of variation (8.92%) demonstrates that the experiment was highly reliable. The experimental validation of quadratic equation was initiated with a medium containing 10.07 g·L^−1^ glucose, 10.07 g·L^−1^ yeast extract powder, 10.00 g·L^−1^ proline, 10 mg·L^−1^ FeSO_4_·7H_2_O, 10 mg·L^−1^ MnSO_4_·4H_2_O, and 1.0 mg·L^−1^ ZnSO_4_·7H_2_O. The experimental values resulted in 11.17 × 10^3^ U·mL^−1^, which was found to be very close to the predicted values of 11.24 × 10^3^ U·mL^−1^.

**Figure 6:**
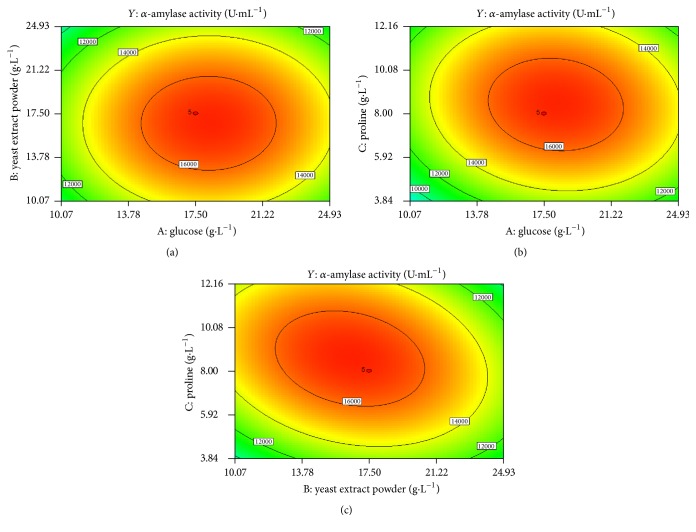
Influence of culture medium component concentrations on extracellular *α*-amylase activity. Two-dimensional contour plots showing the interactions between (a) glucose and yeast extract powder, at a proline concentration of 8 g·L^−1^; (b) glucose and proline at a yeast extract powder concentration of 17.5 g·L^−1^; (c) yeast extract powder and proline at a glucose concentration of 17.5 g·L^−1^.

Figure 6 showed the response surface plots and their corresponding contour plots, which were obtained by the use of Design-Expert software. These representations show that the *α*-amylase activity increased as the concentration of glucose increased from 10.07 to 17.50 g·L^−1^. Because glucose could provide the primary energy for recombinant protein synthesis and cell growth, an inadequate glucose concentration may decrease enzyme production. As shown in Figures 6(a) and 6(c), when the yeast extract powder concentration increased from 10.07 to 17.5 g·L^−1^, the *α*-amylase activity displayed a growth trend. However, further increases in the concentration of yeast extract powder resulted in a downtrend. The organic components of the medium decompose during the fermentation, releasing increasing concentrations of ammonia into the culture medium and increasing its pH [14]. When 17.5 g·L^−1^ yeast extract powder was present in the medium, there was sufficient nitrogen for enzyme production but the pH did not increase to an intolerable value (pH 7.9). However, a higher concentration of yeast extract powder (24.93 g·L^−1^) could increase the pH to 9.0, which might seriously inhibit the synthesis of recombinant protein. As shown in Figures 6(b) and 6(c), the *α*-amylase activity increased as the proline concentration increased from 3.43 to 8.00 g·L^−1^.

The RSM model showed that a medium containing 18.13 g·L^−1^ glucose, 16.48 g·L^−1^ yeast extract powder, 8.55 g·L^−1^ proline, 10 mg·L^−1^ FeSO_4_·7H_2_O, 10 mg·L^−1^ MnSO_4_·4H_2_O, and 1.0 mg·L^−1^ ZnSO_4_·7H_2_O was optimum for the AmyMH production. Validation was carried out under conditions predicted by the model. The result showed that the predicted response for *α*-amylase activity of 1.70 × 10^4^ U·mL^−1^ is quite close to the average of observed experimental values of 1.72 × 10^4^ U·mL^−1^. Thus, the model used in this study is available. Through the combined use of single-factor research and response surface methodology, the *α*-amylase activity obviously increased, which exhibited 8-fold increase compared with that of applying the TM medium.

### 3.6. Maltose Production

**Figure 7:**
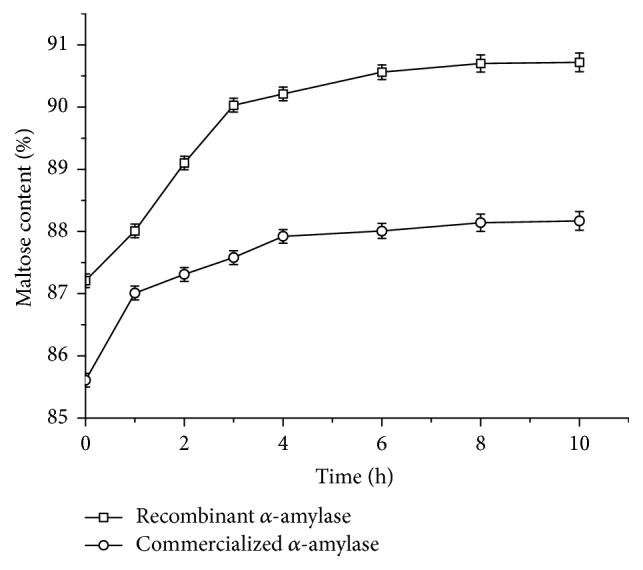
Time profiles of the maltose production reactions performed using recombinant AmyMH and a commercial *α*-amylase. The data represent the average of three independent measurements. The error bars represent the standard deviation.

To investigate the potential use of recombinant AmyMH in the production of maltose, the use of recombinant AmyMH in the starch liquefaction process was compared with the use of commercial *α*-amylase. After the starch liquefaction process, *β*-amylase and pullulanase were added to generate a syrup with maltose as the main component during the first-step saccharification process. As shown in Figure 7, the maltose content of the syrup liquefied with recombinant AmyMH and saccharified with *β*-amylase and pullulanase reached 87.21%. The maltose content obtained using commercial *α*-amylase in the liquefaction process was 85.61%. Maltogenic *α*-amylase was used in second-step saccharification to further improve the maltose content. Figure 7 shows that the maltose content increased during this step. Maximum maltose contents of 90.72% and 88.17% were obtained using recombinant AmyMH and commercial *α*-amylase in liquefaction process, respectively. Considering production of maltose from starch is performed on such a large scale, an increase of 2.55% in maltose content should be considered a substantial improvement. Thus, recombinant AmyMH may be suitable for use in the large-scale production of maltose syrups.

## 4. Discussion

A* Bacillus stearothermophilus* gene encoding maltohexaose-forming, Ca^2+^-independent *α*-amylase was successfully expressed in* E. coli* in a previous report [2]. However, the *α*-amylase activity produced by recombinant* E. coli* was too low to meet the industrial application. In addition,* E. coli* is a kind of pathogenic bacteria and not allowed to be used in food industry. Since nonpathogenic* B. choshinensis* SP3 secretes large amounts of protein with little protease activity, it has been widely used as a host to produce recombinant proteins [5]. In this study, AmyMH from* B. stearothermophilus* was successfully expressed in* B. choshinensis* SP3.

How to produce large amount of recombinant proteins with low cost is one of the key challenges for biochemical workers. Thus, it is one of the most momentous tasks in industrial processes to design and optimize fermentation technology. A limited number of different media have been used for* B. choshinensis* SP3 fermentation. TM [15] and 2SY [16] are the most widely used ones for* B. choshinensis* SP3 fermentation. In this study, the *α*-amylase activity produced by recombinant* B. choshinensis* SP3 using 2SY medium (1250 U·ml^−1^) was only 58.2% of the activity produced using TM medium. Thus, to further improve the production of AmyMH, TM medium was chosen for subsequent optimization experiments.

The temperature and pH of fermentation are important parameters for recombinant protein production in* B. choshinensis* SP3 [6]. A suitable temperature and pH are generally beneficial for both cell growth and enzyme production. In the present study, 33°C and pH 7.5 were found to be suitable for AmyMH production. During the single-factor optimization, the glucose and yeast extract powder were found to be the best carbon and nitrogen source, respectively. Glucose could be efficiently utilized by recombinant* B. choshinensis* SP3 and played significant positive effect on production of recombinant protein. It was consistent with a previous report [6]. The nitrogen sources in the initial medium (10 g·L^−1^ polypeptone, 5 g·L^−1^ meat extract, and 2 g·L^−1^ yeast extract) were costly for recombinant protein production. In the present study, a relatively small quantity of nitrogen sources was used. Polypeptone was reported to stimulate the protein production in a previous study [17]. However, yeast extract powder rather than other nitrogen sources was found to obviously enhance the production of AmyMH in the present study. Yeast extract powder obtained by the autolysis of grown yeasts is a rich source of amino acids, peptides, vitamins, and nucleotides. It might enhance the viability of* B. choshinensis* SP3 cells and improve metabolite production. In addition, due to the relatively available raw materials and simple production process, the cost of yeast extract powder is lower than that of polypeptone. So, yeast extract powder is considered as a suitable nitrogen source for the production of AmyMH.

Many reports have been focused on the enhancement of enzyme production from microorganisms caused by the addition of amino acids to the culture medium. Miyashiro et al. [18] reported that glycine and L-isoleucine could improve extracellular protein production by* Bacillus brevis* number 47. The modes of action for these improvement were different. Isoleucine stimulated the synthesis of extracellular and intracellular proteins, while glycine helped stimulate protein excretion [18]. In this study, the addition of proline was found to remarkably improve the production of recombinant AmyMH in* B. choshinensis* SP3. In addition, better cell growth and slower consumption of glucose (Figures 4(a) and 4(b)) suggested that proline might be a source of energy for recombinant* B. choshinensis* SP3 cells during fermentation. However, supplementation with glucose or proline when glucose was depleted produced only a slight improvement in the production of recombinant *α*-amylase (Figures 4(c) and 4(d)). Further flow cytometric analysis showed that proline could maintain the integrity and well-being of the cells. It has been reported that proline could protect membranes from damage and stabilize the structures and activities of proteins and enzymes in plants cells [19]. In this study, the addition of proline might display similar effects on* B. choshinensis* SP3 cells, and the improved state of cells might be beneficial for the production of AmyMH in* B. choshinensis* SP3. The underlying mechanism will be further explored. In addition, the production of other recombinant proteins in the* B. choshinensis* SP3 system, such as maltogenic amylase, sucrose isomerase, and *α*/*β*-CGTase, could also be improved through the addition of proline to the culture medium.

When AmyMH was used in the production of maltose, the maximum maltose content, 90.72%, was higher than that obtained using commercial *α*-amylase. It has been reported that starch hydrolysates consisting of glucose oligomers with odd numbers of glucose monomers are poor substrates for maltose production [10]. The use of AmyMH in the liquefaction of starch could produce maltohexaose, which contains an even number of glucose monomers, as the main component of starch hydrolysate [2]. However, when commercial *α*-amylase was used in the liquefaction process, a larger amount of glucose oligomers with odd numbers of glucose monomers may have been present in the starch hydrolysate. Thus, the use of AmyMH in the production of maltose might be beneficial.

To the best of our knowledge, this is the first report of the expression of maltohexaose-forming *α*-amylase in* B. choshinensis* SP3 and proline addition enhancing recombinant protein production in a* B. choshinensis* SP3 expression system. Furthermore, the enhanced production of recombinant proteins caused by proline addition might be a universal phenomenon. The *α*-amylase activity was increased 8-fold compared to those obtained with the initial medium. As pH and dissolved oxygen cannot be online controlled in shake-flask fermentation, the fermentation of recombinant* B. choshinensis* SP3 will be initiated in a 3 L fermenter for further study in future. The recombinant *α*-amylase produced in this expression system was used in the production of maltose on a laboratory scale, with 15% (m/v) corn starch as the substrate. The maximum maltose content reached 90.72%, which could be classified as an extremely high maltose syrup. Thus, the recombinant AmyMH may be useful for the industrial production of extremely high maltose syrups.

## Supplementary Material

The effects of pH and temperature on α-amylase activity of AmyMH were investigated (Figure S1). The results showed that the optimal pH and temperature were 5.5 and 70 °C, respectively. The kinetic parameters of AmyMH were also detected (Table S1). As shown in Table S1, the Km and kcat values of AmyMH were 3.7±0.2 g/L and 14.0±0.3×10^2^ min^−^^1^, respectively.

## Figures and Tables

**Table 1:** Primers used in this study.

Primers	Sequence (5′→ 3′)^a^
pNCMO2-F	AA**CTGCAG**CCCCGTTCAAT
pNCMO2-R	CCC**AAGCTTA** CGGCCAGGCC ACCAG

^a^Restriction enzyme recognition sites are presented in boldface and underlined.

**Table 2:** Experimental plan and corresponding *α*-amylase activity.

Run order	Glucose (g·L^−1^)		Yeast extract powder (g·L^−1^)		Proline (g·L^−1^)		*α*-Amylase activity (×10^3^ U·mL^−1^)	
	Code A	A	Code B	B	Code C	C	Predicted	Actual
1	1	24.93	−1	10.07	1	12.16	10.03	10.18 ± 0.25
2	−1	10.07	1	24.93	−1	3.84	6.50	7.00 ± 0.21
3	0	17.50	0	17.50	0	8.00	16.89	17.00 ± 0.45
4	0	17.50	0	17.50	−1.68	1.00	7.67	7.15 ± 0.22
5	0	17.50	0	17.50	0	8.00	16.89	16.90 ± 0.38
6	1	24.93	−1	10.07	−1	3.84	8.85	8.00 ± 0.21
7	0	17.50	0	17.50	0	8.00	16.89	17.00 ± 0.45
8	1	24.93	1	24.93	−1	3.84	8.85	7.90 ± 0.22
9	1	24.93	1	24.93	1	12.16	6.66	6.35 ± 0.21
10	−1	10.07	−1	10.07	1	12.16	10.20	11.50 ± 0.31
11	0	17.50	0	17.50	0	8.00	16.89	16.70 ± 0.41
12	0	17.50	−1.68	5.00	0	8.00	9.82	8.72 ± 0.21
13	0	17.50	0	17.50	0	8.00	16.89	16.92 ± 0.43
14	0	17.50	0	17.50	1.68	15.00	9.54	9.20 ± 0.25
15	0	17.50	1.68	30.00	0	8.00	7.69	8.30 ± 0.23
16	−1	10.07	1	24.93	1	12.16	6.48	6.50 ± 0.21
17	−1	10.07	−1	10.07	−1	3.84	5.35	6.00 ± 0.22
18	1.68	30.00	0	17.50	0	8.00	8.69	9.50 ± 0.32
19	−1.68	5.00	0	17.50	0	8.00	6.59	5.30 ± 0.17

**Table 3:** Effects of proline on the expression of heterologous proteins by *B. choshinensis* SP3.

Enzymes	Proline (g·L^−1^)	DCW (g·L^−1^)	Enzyme activity (U·ml^−1^)
Maltogenic amylase	0	4.0 ± 0.2	2519 ± 56
	10	5.5 ± 0.3	4462 ± 89
Sucrose isomerase	0	4.2 ± 0.2	10 ± 1
	10	5.7 ± 0.3	51 ± 3
*α*/*β*-CGTase	0	4.1 ± 0.2	98 ± 8
	10	5.4 ± 0.3	150 ± 14

**Table 4:** ANOVA analysis of the response surface quadratic model.

Source	Sum of squares	Degree of freedom	Mean square	*F* value	*P* value Prob > *F*
Model	3.256*E* + 008	9	3.618*E* + 007	42.72	<0.0001
A	5.290*E* + 006	1	5.290*E* + 006	6.25	0.0339
B	5.462*E* + 006	1	5.462*E* + 006	6.45	0.0317
C	6.028*E* + 006	1	6.028*E* + 006	7.12	0.0257
AB	631.44	1	631.44	7.456*E* − 004	0.9788
AC	2.380*E* + 006	1	2.380*E* + 006	2.81	0.1280
BC	1.183*E* + 007	1	1.183*E* + 007	13.97	0.0046
A^2^	1.457*E* + 008	1	1.457*E* + 008	171.99	<0.0001
B^2^	1.128*E* + 008	1	1.128*E* + 008	133.14	<0.0001
C^2^	1.222*E* + 008	1	1.222*E* + 008	144.24	<0.0001
Residual	7.622*E* + 006	9	8.469*E* + 005		
Lack of fit	7.562*E* + 006	5	1.512*E* + 006	100.29	
Pure error	60320.00	4	15080.00		
Cor total	3.332*E* + 008	18			

*R* ^2^ = 0.9771, adjusted *R*^2^ = 0.9543, predicated *R*^2^ = 0.8243, adequate precision = 17.284, and coefficient of variation = 8.92%.
