# Supplementary material for: Efficient Expression of Maltohexaose-Forming α-Amylase from Bacillus stearothermophilus in Brevibacillus choshinensis SP3 and Its Use in Maltose Production
Source: Biomed Res Int. 2017 Nov 9;2017:5479762. doi: 10.1155/2017/5479762 (PMC5700550; doi:10.1155/2017/5479762)
Supplement: Supplementary file 1 — The effects of pH and temperature on α-amylase activity of AmyMH were investigated (Figure S1). The results showed that the optimal pH and temperature were 5.5 and 70 °C, respectively. The kinetic parameters of AmyMH were also detected (Table S1). As shown in Table S1, the Km and kcat values of AmyMH were 3.7±0.2 g/L and 14.0±0.3×102 min−1, respectively. [file 5479762.f1.pdf]

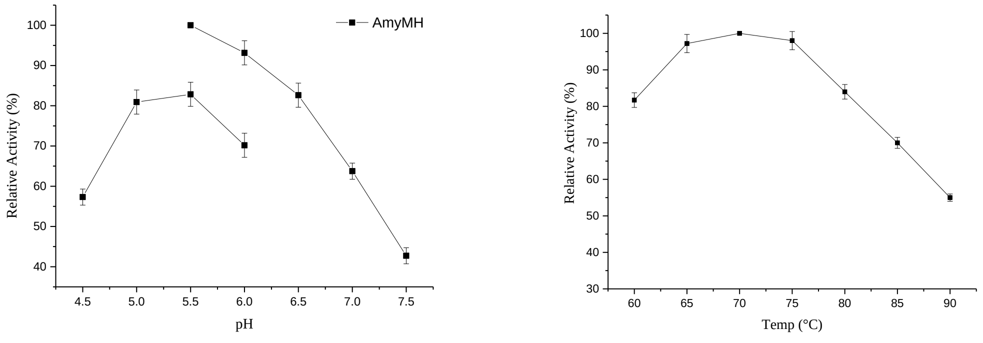

(a) (b)

Fig. S1 The effects of pH (a) and temperature (b) on AmyMH activity

The pH optimum of the recombinant enzyme was determined by measuring its activity across a pH range of 4.5 to 7.5, in 0.5 pH-unit increments, using citrate buffer (pH 4.5 to 6.0) and phosphate buffer (pH 6.0 to 7.5). The temperature dependence of recombinant enzyme activity was measured between 60 and 95 °C. At each temperature, the buffer and substrate were pre-incubated for 10 min before initiating the assay with enzyme. Each reaction was allowed to proceed for 5 min.

Table S1 Kinetic parameters and specific activities of AmyMH

Enzyme	Km (g/L)	kcat ($\times10^2$ min^{-1})	kcat/Km ($\times10^2$ g·L^{-1}·min^{-1})	Specific activity ($\times10^4$ U·mg^{-1})
AmyMH	3.7±0.2	14.0±0.3	3.8±0.2	5.8±0.2

The kinetic parameters (K_m, V_{max}, k_{cat} and k_{cat}/K_m) of AmyMH were determined in 50 mM sodium phosphate buffer (pH 6.0) at 70 °C. Assays performed with purified enzyme contained soluble starch at concentrations ranging from 1 to 20 g·L^{-1}. V_{max} and K_m values were estimated by fitting the initial rate data to the Michaelis-Menten equation using nonlinear regression with GraphPad Prism software (GraphPad Software Inc., San Diego, CA).
